# Antipsychotic Drugs Inhibit Platelet Aggregation via P2Y_**1**_ and P2Y_**12**_ Receptors

**DOI:** 10.1155/2016/2532371

**Published:** 2016-03-16

**Authors:** Chang-Chieh Wu, Fu-Ming Tsai, Mao-Liang Chen, Semon Wu, Ming-Cheng Lee, Tzung-Chieh Tsai, Lu-Kai Wang, Chun-Hua Wang

**Affiliations:** ^1^Department of Surgery, Tri-Service General Hospital, National Defense Medical Center, Taipei 114, Taiwan; ^2^Department of Research, Taipei Tzuchi Hospital, The Buddhist Tzuchi Medical Foundation, New Taipei City 231, Taiwan; ^3^Department of Life Science, Chinese Culture University, Shih Lin, Taipei 111, Taiwan; ^4^Department of Microbiology, Immunology and Biopharmaceuticals, National Chiayi University, Chiayi 600, Taiwan; ^5^Radiation Biology Core Laboratory, Institute for Radiological Research, Chang Gung University/Chang Gung Memorial Hospital, Linkou, Taoyuan 333, Taiwan; ^6^Department of Dermatology, Taipei Tzuchi Hospital, The Buddhist Tzuchi Medical Foundation, New Taipei City 231, Taiwan

## Abstract

Antipsychotic drugs (APDs) used to treat clinical psychotic syndromes cause a variety of blood dyscrasias. APDs suppress the aggregation of platelets; however, the underlying mechanism remains unknown. We first analyzed platelet aggregation and clot formation in platelets treated with APDs, risperidone, clozapine, or haloperidol, using an aggregometer and rotational thromboelastometry (ROTEM). Our data indicated that platelet aggregation was inhibited, that clot formation time was increased, and that clot firmness was decreased in platelets pretreated with APDs. We also examined the role two major adenosine diphosphate (ADP) receptors, P2Y_1_ and P2Y_12_, play in ADP-mediated platelet activation and APD-mediated suppression of platelet aggregation. Our results show that P2Y_1_ receptor stimulation with ADP-induced calcium influx was inhibited by APDs in human and rats' platelets, as assessed by* in vitro* or* ex vivo* approach, respectively. In contrast, APDs, risperidone and clozapine, alleviated P2Y_12_-mediated cAMP suppression, and the release of thromboxane A2 and arachidonic acid by activated platelets decreased after APD treatment in human and rats' platelets. Our data demonstrate that each APD tested significantly suppressed platelet aggregation via different mechanisms.

## 1. Introduction

Antipsychotic drugs (APDs) are used clinically to ease the symptoms of schizophrenia; however, they can also alter certain functions of the immune system, which often result in negative side effects. APDs affect serum levels of interleukin- (IL-) 1*β*, IL-2, IL-6, IL-8, and IL-10 and interferon- (IFN-) *γ*, as well as the expression of the receptors for IL-2 and IL-6 in patients [1–5]. Atypical APDs, such as clozapine and risperidone, inhibit Th1 differentiation and decrease T-cell production of IFN-*γ* [6, 7]. However, haloperidol, a typical APD, decreases Th2 differentiation and inhibits T-cell production of IL-4 [7]. Risperidone modulates chemokine and cytokine release from dendritic cells, which are tasked with regulating Th_1_/Th_2_ differentiation [8]. Additional reports show changes in the phagocytic abilities of macrophages and neutrophils treated with the APDs, risperidone, clozapine, or haloperidol [9, 10].

Platelets are small anucleate cell fragments (1–3 *μ*m) released by megakaryocytes (50–100 *μ*m) via cytoplasmic extensions called proplatelets [11]. Platelets are the blood cells responsible for maintaining hemostasis and play an important role in thrombosis, wound healing, atherosclerosis, inflammation, immunity, and tumor metastasis [12, 13]. The primary physiological function of platelets is to form hemostatic thrombi during hemorrhaging. Platelet aggregation is regulated by the production of thromboxane and prostaglandin (PG) that originates from platelets, blood vessels, and other tissues [14–18]. The action and physiological roles of thromboxane A_2_ (TXA_2_) and prostacyclins are well-established [19–23]. Many different physiological agonists such as coagulation factors (thrombin), hormones (epinephrine), low-molecular-weight substances (serotonin and adenosine diphosphate [ADP]), lipid derivatives (platelet aggregating factor), TXA_2_, and collagen activate platelets. The most established platelet stimulus is ADP, which induces multiple platelet responses and potentiates platelet aggregation [24, 25]. ADP acts on two G protein-coupled receptors, P2Y_1_ and P2Y_12_. The P2Y_1_ receptor is widely expressed throughout the body and couples to Gq, which leads to the activation of phospholipase C*β*, increases cytosolic calcium levels, and activates protein kinase C. The P2Y_12_ receptor couples with Gi to inhibit adenylyl cyclase and activate PI3-kinase [26–29]. Both outside-in signaling from the fibrinogen receptor and inside-out signaling from the P2Y_1_ and P2Y_12_ receptors are necessary for phospholipase A_2_ activation, arachidonic acid release, and thromboxane A_2_ generation in platelets [30].

APDs cause a variety of blood dyscrasias. Numerous reports discuss the risks of adverse hematological effects, such as neutropenia or thrombocytopenia, associated with psychotropic drug usage. For example, schizophrenic patients treated with APDs are more likely to develop cardiovascular diseases [31–33]. Associated cardiovascular diseases may be caused by an interruption of blood platelet activity. Semiz et al. showed that an increase in the mean platelet volume was associated with treating schizophrenic patients with APDs [34]. Despite the fact that APDs affect platelet aggregation* in vitro* [35], the precise mechanism of APD's effect on the aggregative ability of the whole blood still remains unclear. Due to the short lifespan of purified platelets, it is difficult to study the effect of APDs on purified platelets over extended periods of time; therefore, the quick assays used in previous studies [35] may not reflect the clinical syndrome or the long-term effects of APD treatment* in vivo*.

In this study we analyzed the effects of APDs on platelet aggregation and describe the molecular mechanism responsible for these effects. In addition, chronic APD treatment of rats was performed to address the impact of APDs on platelet aggregation in schizophrenic patients treated with APDs over extended periods of time.

## 2. Materials and Methods

### 2.1. Reagents

All chemicals were purchased from Sigma-Aldrich (St. Louis, MO) except risperidone (Janssen-Pharmaceutica, Beerse, Belgium), adenosine 2′,5′-diphosphate (A2P5P, Santa Cruz Biotechnology, Santa Cruz, CA), and clopidogrel (Santa Cruz Biotechnology).

### 2.2. Animals and Drug Treatment

Male Sprague-Dawley rats weighing 350–400 g were used at the beginning of all experiments. The animals were housed in a temperature and humidity-controlled environment with a 12-hour light/dark cycle and had free access to food and water. All experimental procedures were approved by the Taipei Tzuchi Hospital, the Buddhist Tzuchi Medical Foundation Institutional Animal Care and Use Committee. Rats (*n* = 3 per group) were given one of the following treatments: haloperidol (1 mg/kg), clozapine (20 mg/kg), risperidone (1 mg/kg), or saline. Each animal received an intraperitoneal injection once a day for 28 days. These doses were chosen from the literature and corresponded to clinically relevant treatments [36, 37].

### 2.3. Preparation of Human and Rats' Platelets

Human blood was obtained by venipuncture from healthy adults and collected in a vacutainer containing sodium citrate. Informed consent was obtained from all participants in the study, and our study was reviewed and approved by the Taipei Tzuchi Hospital, the Buddhist Tzuchi Medical Foundation Institutional Review Board. Blood from rats, anesthetized via intramuscular injection of zolitel (30 mg/kg) and xylazine (6 mg/kg), was drawn from the abdominal aorta and collected in a vacutainer containing sodium citrate. Platelet-rich plasma (PRP) was prepared by centrifugation at 180 ×g for 20 minutes. Platelets and platelet poor plasma (PPP) were then obtained by centrifugation for 15 minutes at 1500 ×g. The pellet was resuspended to a density of 2 × 10^8^ platelets/mL in a modified calcium-free Tyrode buffer (138 mM NaCl; 2.7 mM KCl; 1 mM MgCl_2_; 3 mM NaH_2_PO_4_; 10 mM HEPES; 5 mM glucose; 0.2% BSA; and 20 *μ*g/mL apyrase, pH 7.4).

### 2.4. Light Transmission Aggregometry and Rotation Thromboelastometry (ROTEM) Analysis

ADP-induced platelet aggregation was monitored using a light transmittance aggregometer (PAP-8E, Platelet Aggregation Profiler, Bio/Data Corporation, Horsham, PA). Baseline optical density was set using the PPP sample. Aggregation induced by 10 *μ*M ADP was monitored for 6 minutes. For the ROTEM analysis, human blood was collected and treated with 1 × 10^-6 ^M APDs for 1 hour. Three-hundred microliters of APD-treated blood was used for the ROTEM analysis, which was performed according to the manufacturer's instructions using equipment and kits provided by TEM International GmbH (Pentapharm, Munich, Germany). We used the kit for extrinsically activated coagulation (EXTEM), which induces clot formation via the extrinsic pathway. The following parameters were measured for the ROTEM: clotting time (CT, the time from the start of the assay to clot formation with an amplitude of 2 mm), clot formation time (CFT, the time from the end of CT (amplitude of 2 mm) to a clot firmness of 20 mm), and maximum clot firmness (MCF, the peak strength of the clot). A10, A15, A20, A25, and A30 correspond to the maximum amplitude of the curve after 10, 15, 20, 25, and 30 minutes, respectively.

### 2.5. Measurement of Cytosolic Free Calcium in Platelets

Chronic APD-treated rat PRP was prepared. Human PRP was treated with various APDs for 1 hour. 3 *μ*M Fura-2AM (Molecular Probes, Invitrogen, Carlsbad, CA) was added to the PRP and incubated at 37°C for 45 minutes. Platelets were centrifuged and resuspended in our modified calcium-free Tyrode buffer. 10 *μ*M ADP-induced calcium responses were measured at 37°C in a multifunctional microplate reader (Infinite F200, Tecon, Durham, NC) with fluorescence excitation set at 340 nm and 380 nm, while emission was set to 510 nm.

### 2.6. Measurement of cAMP Levels in Platelets

Human PRP was treated with various APDs for 1 hour. Isolated platelets from human or chronic APD-treated rats were incubated with 10 *μ*M forskolin and 10 *μ*M ADP at 37°C for 5 minutes. After washing twice with PBS, cells were lysed with 0.1 N HCl, scraped, and collected by centrifugation. Levels of cAMP in the supernatants were determined using a cyclic AMP EIA kit according to the manufacturer's instructions (Cayman Chemical, Ann Arbor, MI).

### 2.7. Measurement of Thromboxane A_2_ (TXA_2_) in Platelets

Human PRP was treated with various APDs for 1 hour. Isolated platelets from human or chronic APD-treated rats were stimulated by adding 3 *μ*M fibrinogen with or without 10 *μ*M ADP at 37°C for 3 minutes. The reaction was stopped by quickly freezing the sample in a dry ice-ethanol bath. After thawing at room temperature, the samples were centrifuged at 3000 ×g for 10 minutes at 4°C. The supernatants were collected and used to measure the content of thromboxane B_2_, the stable metabolite of TXA_2_, using an enzyme immunoassay (EIA) according to the manufacturer's instructions (Cayman Chemical).

### 2.8. Measurement of Arachidonic Acid Liberation in Platelets

Human PRP was treated with various APDs for 1 hour. Isolated platelets from human or chronic APD-treated rats were activated with 10 *μ*M ADP plus fibrinogen at 37°C for 3 minutes. Levels of arachidonic acid in the supernatants were determined using an arachidonic acid ELISA kit (Cusabio Biotech, Wuhan, Hubei, China).

### 2.9. Statistical Analysis

Data are presented as mean ± SD of at least triplicate studies. Statistical analyses were performed using one-way ANOVA with Dunnett's post hoc test for comparison of more than two groups. A *p* value < 0.05 was considered statistically significant.

## 3. Results

### 3.1. Effect of APDs on Platelet Aggregation

**Figure 1:**
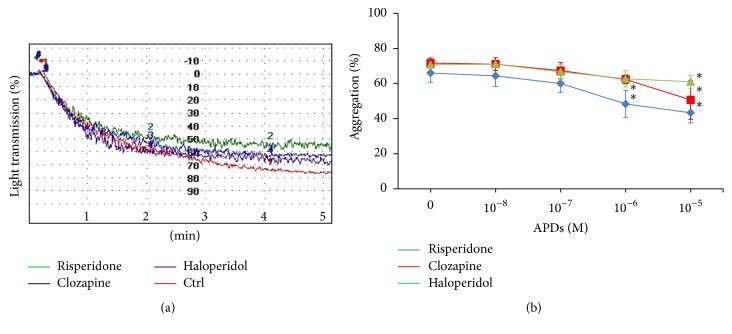
The effect of APDs on ADP-induced platelet aggregation in human platelets. PRPs were preincubated with 1 × 10^−6^ M (a) or indicated concentration (b) of APDs before stimulation with 10 *μ*M ADP. This is a representative graph illustrating the aggregometer readings from one of three independent experiments (a). Experimental results are summarized as mean (±SD) percentage of aggregation in PRP (b). ^*∗*^ *p* < 0.05.

To examine the effect of APDs on platelet aggregation, PRPs were pretreated with APDs for 1 hour and platelet aggregation was performed. In agreement with previous studies [35], ADP-induced aggregation was suppressed by risperidone, clozapine, and haloperidol (Figure 1(a)). Treatment of the human PRP with the different doses of APDs resulted in a concentration-dependent inhibition of ADP-induced platelet aggregation* in vitro* (Figure 1(b)). ROTEM performed on whole blood samples provided information on the contribution of fibrinogen and platelets to clot formation. Results from these experiments were meaningful given that the experimental conditions were similar to physiological conditions [38]. To monitor the effect of APDs on platelet aggregation* in vivo*, whole blood cells were pretreated with APDs for 1 hour and ROTEM analysis was performed. Results from an extrinsically activated assay using recombinant tissue factor (EXTEM) indicated no significant differences in the coagulation time between control blood and APD-treated blood. The clot formation time was significantly increased in blood treated with risperidone or clozapine (Table 1). The amplitude at 5 to 30 minutes and the MCF were significantly lower in blood treated with each APD compared with the control group (Table 1). These results showed that aggregation and blood clot strength decreased in blood treated with APDs.

**Figure 2:**
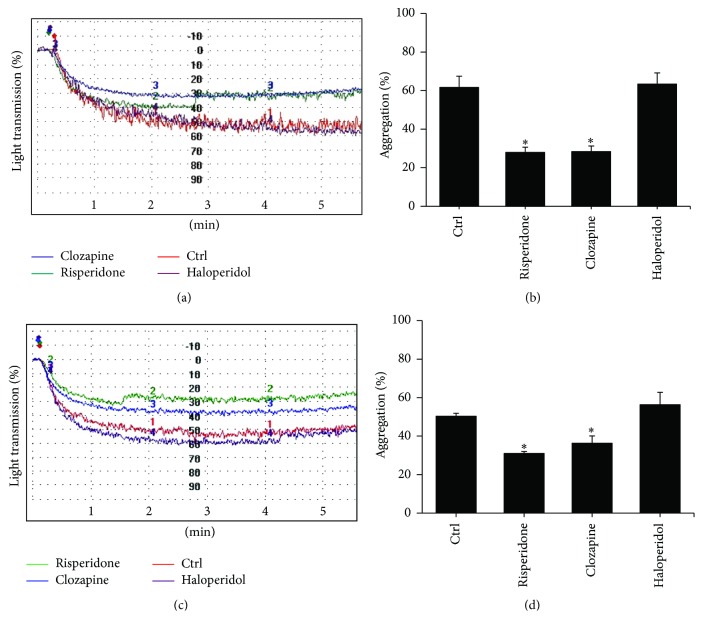
The effect of chronic APDs on ADP-induced platelet aggregation in platelets from rats. Rats were treated with haloperidol (1 mg/kg), clozapine (20 mg/kg), risperidone (1 mg/kg), or saline for 7 days (a and b) or 28 days (c and d) as described in Section 2. PRPs were prepared and stimulated with 10 *μ*M ADP. These are representative graphs illustrating the aggregometer readings from one of three independent experiments (a and c). Experimental results are summarized as mean (±SD) percentage of aggregation in PRPs (b and d). ^*∗*^ *p* < 0.05.

To realize the chronic effect of APD on platelet aggregation, we analyzed chronic APD-treated rats to observe possible effects on platelet aggregation. After stimulation with ADP, rats chronically treated with risperidone and clozapine exhibited significantly reduced platelet aggregation by 54.6% and 54.1% at day 7 and by 38.4% and 27.8% at day 28, respectively (Figure 2). Haloperidol exhibited no effect on ADP-induced platelet aggregation in comparison with the control group.

### 3.2. Effect of APDs on P2Y_1_ and P2Y_12_ in Platelets

**Figure 3:**
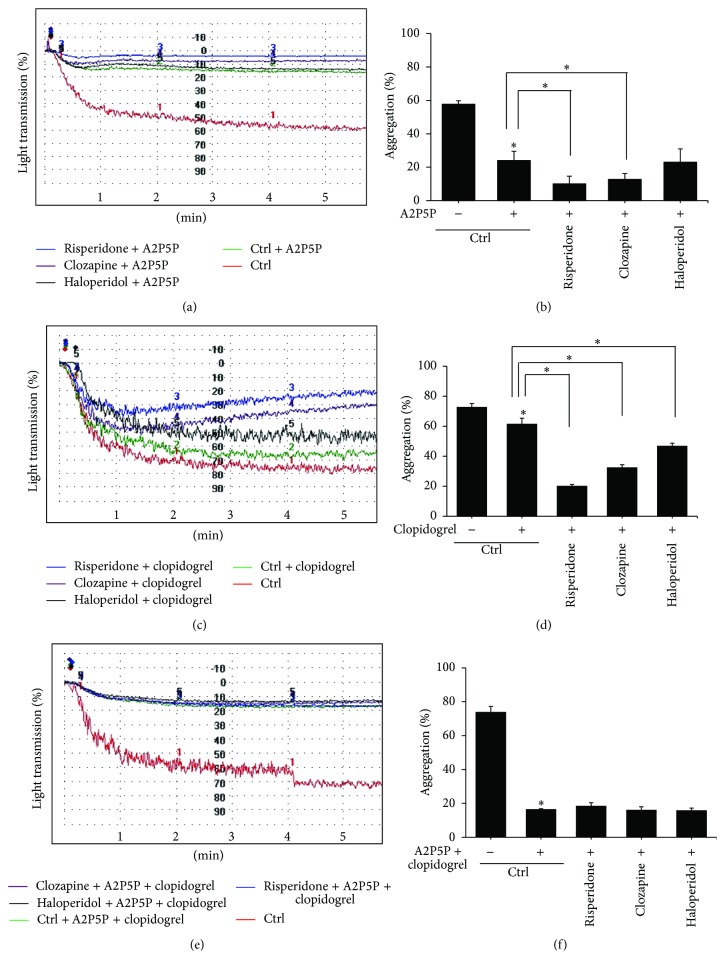
The effect of P2 receptor antagonists on APDs-mediated platelet aggregative suppression. PRPs were preincubated with 1 × 10^−6^ M APDs before stimulation with 10 *μ*M ADP alone or in the presence of 1 mM A2P5P (a and b), 30 *μ*M clopidogrel (c and d), or both A2P5P and clopidogrel (e and f). These are representative graphs illustrating the aggregometer readings from one of three independent experiments (a, c, and e). Experimental results are summarized as mean (±SD) percentage of aggregation in PRPs (b, d, and f). ^*∗*^ *p* < 0.05.

ADPs play a central role in regulating platelet function by activating the G-protein-coupled receptors, P2Y_1_ and P2Y_12_. We first determined the expression of P2Y_1_ and P2Y_12_ in platelet treated with APDs. No significant change in P2Y_1_ and P2Y_12_ expression was observed in platelet treated with each APD (data not shown). We then examined the P2 receptor antagonists on APD-mediated suppression of platelet aggregation. Pretreatment of platelets with the P2Y_1_ receptor antagonist A2P5P (1 mM; 3 minutes) abolished ADP-induced platelet aggregation by 58.3%. Pretreatment of 1 × 10^−6^ M risperidone and clozapine further significantly reduced platelet aggregation by 57.3% and 50%, respectively, in platelets preincubated with A2P5P (Figures 3(a) and 3(b)). ADP-induced platelet aggregation was decreased by 17.2% in platelets pretreated with P2Y_12_ receptor antagonist clopidogrel (30 *μ*M; 3 minutes) and reached a range from 34.5 to 73.1% when platelets were coincubated with 1 × 10^−6^ M APDs (Figures 3(c) and 3(d)). None of the three APDs can affect platelet aggregative ability in platelets pretreated with A2P5P and clopidogrel indicated APDs would inhibit platelet aggregation via P2Y_1_ and P2Y_12_ receptors (Figures 3(e) and 3(f)).

**Figure 4:**
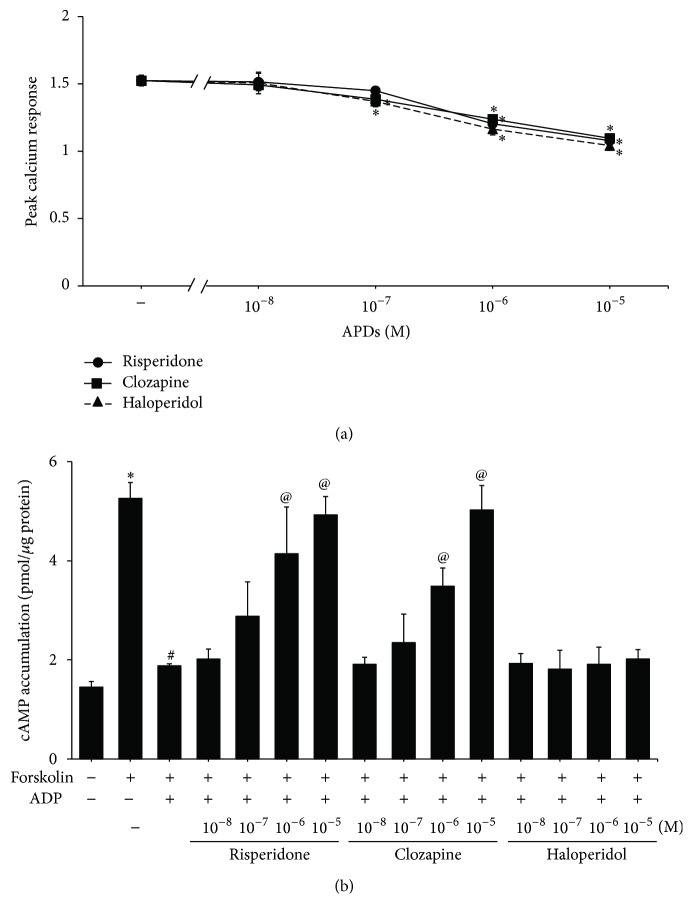
The effect of APDs on P2Y_1_ and P2Y_12_ in human platelets. (a) PRPs were treated with the indicated APD concentrations for 1 hour. They were then incubated with Fura-2AM at 37°C. Platelets were harvested and ADP-induced calcium responses were measured. The peak responses were compared with those in nonpretreated control rats. ^*∗*^ *p* value < 0.05. (b) PRPs were treated with the indicated APD concentrations for 1 hour. Isolated platelets were then incubated with 10 *μ*M forskolin and 10 *μ*M ADP at 37°C. Cell lysates were prepared and the levels of cAMP were measured using an enzyme immunoassay. Data are represented as the mean ± SEM (*n* = 3). ^*∗*^ *p* value < 0.05 for the control group compared to control group treated with forskolin. ^#^ *p* value < 0.05 for the control group treated with forskolin and ADP compared to the control group treated with forskolin only. ^@^ *p* value < 0.05 for cells treated with APDs followed by forskolin and ADP stimulation compared to cells stimulated with forskolin and ADP alone.

To further dissect the mechanisms underlying the effect of APDs on agonist-induced platelet aggregation, we evaluated the effect of APDs on ADP-induced calcium influx and cAMP suppression in PRP.  Figure 4(a) shows the reduction in calcium responsiveness in PRP pretreated with APDs (10^−7^–10^−5^ M APD). We also determined whether platelet P2Y_12_ receptor-mediated responses are affected by APDs. We confirmed that forskolin-stimulated cAMP production was inhibited by ADP. This inhibition was reversed with 10^−6^–10^−5^ M risperidone or clozapine pretreatment, but not with haloperidol (Figure 4(b)). These results suggest that some APDs are effective in the inhibition of the P2Y_1_ receptor response.

**Figure 5:**
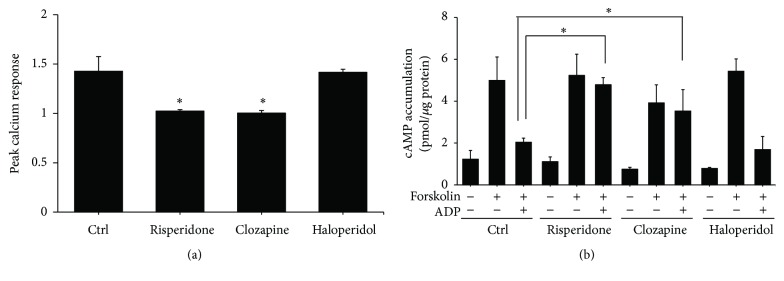
The effect of chronic APDs on P2Y_1_ and P2Y_12_ in platelets from rats. Chronic APD-treated PRPs were produced by treating rats with the indicated concentration of APDs as described in Section 2. (a) Platelets were harvested at day 28 and ADP-induced calcium responses were measured. The peak responses were compared with those in nonpretreated ADP platelets. ^*∗*^ *p* value < 0.05 for cells treated with chronic APDs followed by ADP stimulation compared to control cells stimulated with ADP alone. (b) Isolated platelets were harvested at day 28 and then incubated with 10 *μ*M forskolin and 10 *μ*M ADP at 37°C. Cell lysates were prepared and the levels of cAMP were measured using an enzyme immunoassay. Data are represented as the mean ± SEM (*n* = 3). ^*∗*^ *p* value < 0.05 for cells treated with chronic APDs followed by forskolin and ADP stimulation compared to control cells stimulated with forskolin and ADP alone.

The ADP-induced calcium influx was decreased with long-term treatment of risperidone and clozapine in platelets from rats (Figure 5(a)). After stimulation with forskolin, cAMP was increased in control and all drug treated rat platelets. The increased levels of cAMP were reversed by ADP in control group or haloperidol-treated rats by 59.2% or 68.9%, respectively (Figure 5(b)). Platelets from rats chronically treated with risperidone and clozapine failed to reverse the ADP-mediated cAMP suppression that was induced with the addition of forskolin.

**Figure 6:**
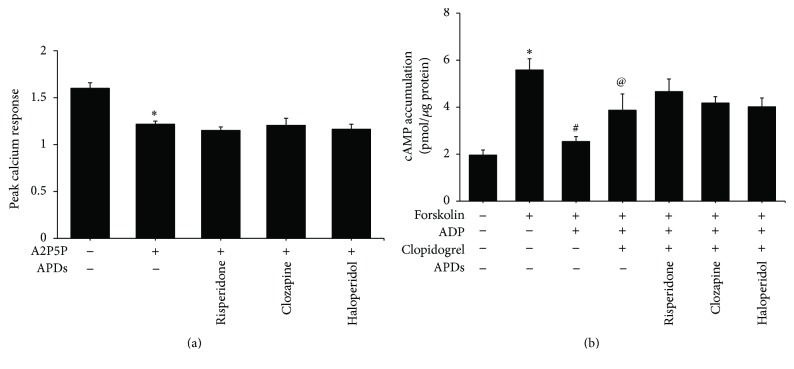
The effect of P2 receptor antagonists on APDs-mediated P2Y_1_ and P2Y_12_ receptors signaling in human platelets. (a) PRPs were pretreated with 1 × 10^−6^ M APDs for 1 hour and then incubated with Fura-2AM at 37°C. Platelets were harvested and then treated with 1 mM A2P5P for 3 min. ADP was added and subsequent calcium responses were measured. ^*∗*^ *p* value < 0.05 for the control group compared to control group treated with A2P5P. (b) PRPs were pretreated with 1 × 10^−6^ M APDs for 1 hour. Isolated platelets were then incubated with 10 *μ*M forskolin, 30 *μ*M clopidogrel, and 10 *μ*M ADP at 37°C. Cell lysates were prepared and the levels of cAMP were measured using an enzyme immunoassay. Data are represented as the mean ± SEM (*n* = 3). ^*∗*^ *p* value < 0.05 for the control group compared to control group treated with forskolin. ^#^ *p* value < 0.05 for the control group treated with forskolin and ADP compared to the control group treated with forskolin only. ^@^ *p* value < 0.05 for cells treated with clopidogrel followed by forskolin and ADP stimulation compared to cells stimulated with forskolin and ADP alone.

A2P5P significantly inhibited ADP-induced calcium influx in platelets (Figure 6(a)) and clopidogrel alleviated ADP-mediated cAMP suppression (Figure 6(b)). None of the three APDs affected calcium influx or cAMP production in platelets pretreated with A2P5P or clopidogrel, respectively (Figures 6(a) and 6(b)). The observation further confirmed APDs-mediated platelet aggregative suppression through P2Y_1_ and P2Y_12_ receptors.

### 3.3. Effect of APDs on ADP-Induced TXA_2_ and Arachidonic Acid Production in Platelets

**Figure 7:**
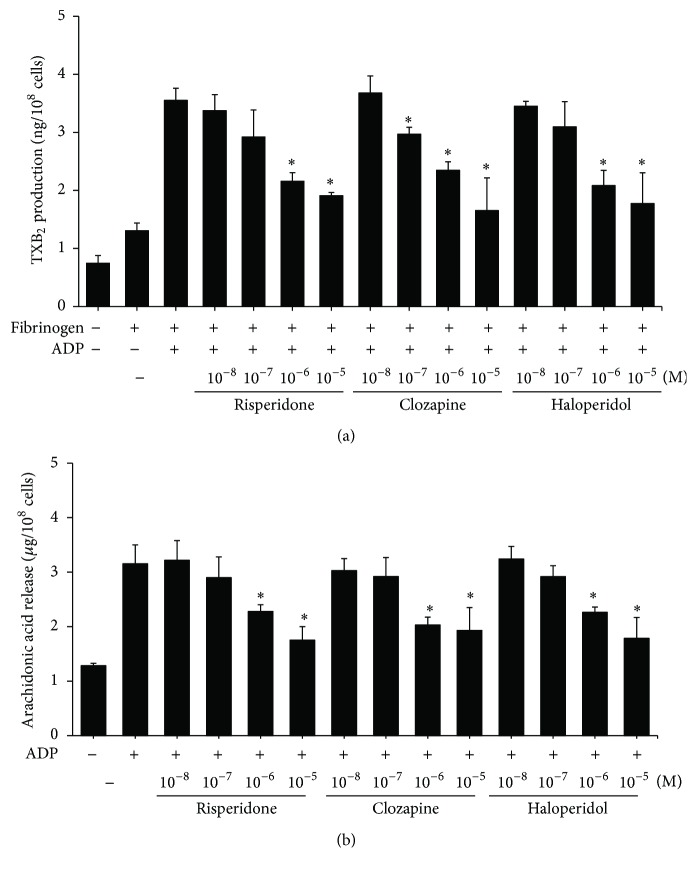
The effect of APDs on APD-induced TXA_2_ and arachidonic acid production in human platelets. PRPs were treated with the indicated concentrations of APDs for 1 hour. Isolated platelets were then stimulated with 3 *μ*M fibrinogen and 10 *μ*M ADP at 37°C for 3 minutes. Reactions were stopped and the content of thromboxane B_2_ (a) or arachidonic acid (b) was measured using an enzyme immunoassay. Data represent the mean ± SEM (*n* = 3). ^*∗*^ *p* value < 0.05.

TXA_2_, which is produced by activated platelets, stimulates the activation of new platelets and increases platelet aggregation. We examined the effect of APDs on TXA_2_ production in platelets treated with fibrinogen. APDs inhibited ADP-induced TXB_2_, the stable metabolite of TXA_2_, in a dose-dependent manner (Figure 7(a)). Arachidonic acids are converted to TXA_2_ by activated phospholipase A_2_ through the cyclooxygenase pathway. To investigate whether APDs block the release of arachidonic acid, or its subsequent conversion to thromboxane A_2_, we evaluated the release of arachidonic acid in activated platelets pretreated with APDs. As shown in Figure 7(b), APDs inhibited the ADP-induced release of arachidonic acid in a dose-dependent manner.

**Figure 8:**
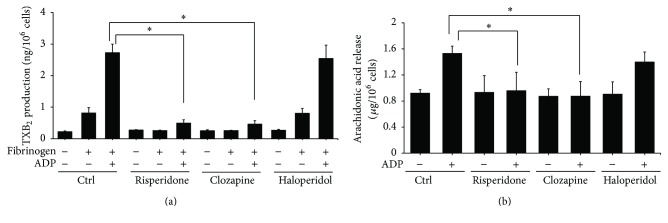
The effect of chronic APDs on APD-induced TXA_2_ and arachidonic acid production in platelets from rats. Chronic APD-treated PRPs were produced by treating rats with the indicated concentration of APDs as described in Section 2. Isolated platelets were harvested at day 28 and then stimulated with 3 *μ*M fibrinogen and 10 *μ*M ADP at 37°C for 3 minutes. Reactions were stopped and the content of thromboxane B_2_ (a) or arachidonic acid (b) was measured using an enzyme immunoassay. Data represent the mean ± SEM (*n* = 3). ^*∗*^ *p* value < 0.05 for cells treated with chronic APDs followed by fibrinogen and ADP stimulation compared to control cells stimulated with fibrinogen and ADP alone.

We also studied the influence of chronic APD treatment on the production of TXA_2_ and arachidonic acid in platelets from rats. As shown in Figure 8(a), there is a significant decrease of TXA_2_ in fibrinogen- and ADP-stimulated platelets of rats treated chronic risperidone and clozapine as compared to control and chronic haloperidol-treated animals. The release of arachidonic acid of ADP-induced platelets was also inhibited in risperidone- or clozapine-treated rats (Figure 8(b)).

## 4. Discussion

Although the effect of APDs on platelet aggregation* in vitro* is well-documented [35], little data is available regarding their role in decreasing platelet function. A whole blood thromboelastometry can be used to monitor physiological signals of platelets and light transmission aggregometry can be used to obtain information about the features of platelet functions by stimulating different platelet activation pathways with different agonists. In the present study, we confirmed that APD treatment reduced platelet aggregative abilities using aggregometry and ROTEM analysis. Our results offer data supporting a molecular mechanism whereby APDs suppress ADP receptor-mediated activation. APDs inhibited P2Y_1_ activation that is normally induced by ADP, and the P2Y_12_ receptor signal pathway was inhibited by risperidone and clozapine, but not by haloperidol. Furthermore, APDs used in this study inhibited fibrinogen receptor-mediated TXA_2_ and arachidonic acid release in activated platelets. Although all higher doses of APDs reduced the aggregative ability of platelets, the serum levels of risperidone, clozapine, or haloperidol in patients were about 1 × 10^−7 ^M, 1 × 10^−6 ^M, and 2 × 10^−8 ^M, respectively [39–41]. Our data suggests that clinically relevant therapeutic doses of haloperidol do not affect platelet function. In addition, chronic haloperidol treatment had no effect on platelet aggregation or the P2Y_1_- and P2Y_12_-mediated responses.

Schizophrenic patients treated with APDs show increased incidence of mortality due to cardiovascular disease and thrombocytopenia (0.1%−2% in APDs-treated patients) [31–33]. Therefore, we hypothesized that APDs may affect the function of platelets. According to a previous study [35] and our results, APDs do decrease platelet aggregation. This indicated that patients treated with APDs may be at risk for increased incidence of hemorrhaging. In order to understand the effects of APDs on platelet function in a clinical setting, platelets purified from schizophrenic patients undergoing long-term APD treatment should be studied to better understand platelet aggregation.

The effects of APDs on platelet aggregation cannot explain the development of thrombocytopenia in patients treated on the long term with APDs. In addition to affecting platelet aggregation, APDs may also decrease platelet formation during differentiation. Platelet formation is divided into two phases. The first phase consists of megakaryocyte maturation and development, which requires the megakaryocyte-specific growth factor, thrombopoietin. The second phase involves platelet generation via the initial remodeling of the megakaryocyte cytoplasm into proplates and later into preplatelets, which then undergo subsequent fission events to generate platelet fragments [42]. Growing evidence suggests that the megakaryocyte cytoskeleton is the principal machinery responsible for platelet production. Mutations in the* MYH9* gene, which reduces myosin II activity, are implicated in related macrothrombocytopenias [43]. Studies show that* MYH9* mutations are associated with proplatelet formation, which is mediated by disruption of the Rho/ROCK and Rho kinase-myosin light chain 2 pathways, thus leading to decreased numbers of circulating platelets [44, 45]. Interestingly, according to our proteomics analysis, C6 glial cells treated with APDs increased expression of RhoGDI1, a downregulator of Rho family GTPases. Increased RhoA was observed in C6 cells treated with APDs for 6 days (data not shown). Further studies are needed to answer whether increased Rho activity in cells treated with APDs would decrease the formation of proplatelets.

To date, the receptor-mediated network of signal transduction mechanisms underlying the various therapeutic properties of most APDs is still not known. APDs are classified as typical or atypical, according to the receptors they bind. First-generation APDs, known as typical APDs, including haloperidol, bind mainly to the dopamine D2 receptor rather than to other receptors [46, 47]. Most second-generation drugs, known as atypical APDs, such as risperidone and clozapine, bind not only to dopamine D2 receptors, but also to type II serotonin receptors, dopamine D3 receptor, and dopamine D4 receptor [48, 49]. The mechanism for these receptors bound by atypical APDs involving in P2Y_12_ receptor pathway is still unknown. Elucidation of APD-mediated modulation of platelets will be difficult due to the complex binding profile of APDs. For example, clozapine interferes with the binding of dopamine, GABAB, and 5-HT receptors but also acts as an agonist on 5-HT1A receptors. Regardless of the receptors binding APDs, our current results argue that platelet aggregative abilities are suppressed in platelets treated with APDs. Furthermore, the P2Y_1_ receptor pathway was inhibited by risperidone, clozapine, and haloperidol, while the P2Y_12_ receptor pathway was inhibited by risperidone and clozapine. In total, our results suggest that APDs affect different targets to inhibit platelet aggregation.

## 5. Conclusions

Our data demonstrate that risperidone, clozapine, and haloperidol suppressed platelet aggregation via different targets. Risperidone or clozapine inhibited platelet aggregation via P2Y_1_ and P2Y_12_, whereas haloperidol affected P2Y_1_ only. In addition, decreased platelet aggregability was observed in chronic risperidone- and clozapine-treated rats; therefore, this may be the cause of the hematologic side effects associated with APD treatment.

## Figures and Tables

**Table 1:** ROTEM measurements of EXTEM in blood treated with APDs (*n* = 3).

Parameters	Control	Risperidone	Clozapine	Haloperidol
	Coagulation activation and clot polymerization parameters			
Coagulation time (seconds)	60 ± 4.6	71.3 ± 25.6	69.7 ± 26	53.3 ± 25.1
Clot formation time (seconds)	115.3 ± 7.6	171.7 ± 19.3^*∗*^	169 ± 11.1^*∗*^	151.3 ± 33.2
*α*-angle (degree)	68.7 ± 0.6	58 ± 2.6^*∗*^	58.3 ± 1.5^*∗*^	61.3 ± 5.8

	Clot firmness parameters (mm)			
Amplitude at 5 min (A5)	45.3 ± 1.5	30.3 ± 0.6^*∗*^	30.7 ± 0.6^*∗*^	35 ± 5.2^*∗*^
A10	58 ± 1	42.3 ± 0.6^*∗*^	43 ± 1^*∗*^	47 ± 5.3^*∗*^
A15	62.3 ± 0.6	47.7 ± 0.6^*∗*^	48 ± 1^*∗*^	52.3 ± 4.2^*∗*^
A20	64.3 ± 0.6	50.7 ± 0.6^*∗*^	51.7 ± 1.5^*∗*^	55.3 ± 4.2^*∗*^
A25	64.3 ± 0.6	52.7 ± 0.6^*∗*^	53.3 ± 2.1^*∗*^	57 ± 3.6^*∗*^
A30	63 ± 1	53.7 ± 0.6^*∗*^	54.3 ± 2.1^*∗*^	57.7 ± 3.1^*∗*^
Maximum clot firmness (MCF)	64.7 ± 0.6	54.3 ± 0.6^*∗*^	54.7 ± 2.5^*∗*^	57.7 ± 3.1^*∗*^

ROTEM: rotation thromboelastometry; EXTEM: extrinsically activated assay using recombinant tissue factor.

The data are expressed as the median (interquartile range). ^*∗*^ *p* < 0.05 compared with the control group.
